# Determining the Temperature-Dependent Air–Water Partitioning of Ether- and Thioether-Alcohol Perfluoroalkyl and Polyfluoroalkyl Substances Using a Modified Static Headspace Method

**DOI:** 10.1021/acs.est.4c11447

**Published:** 2025-07-28

**Authors:** Viktória Licul-Kucera, Annemarie P. van Wezel, Hans Peter H. Arp, Thomas L. ter Laak

**Affiliations:** † Institute for Biodiversity and Ecosystem Dynamics, University of Amsterdam, Science Park 904, 1098 XH Amsterdam, The Netherlands; ‡ 72989 Norwegian Geotechnical Institute, P.O. Box 3930, Ullevål Stadion, N-0806 Oslo, Norway; § Norwegian University of Science and Technology, 7024 Trondheim, Norway; ∥ KWR Water Research Institute, P.O. Box 1072, 3430 BB Nieuwegein, The Netherlands

**Keywords:** PFAS, air−water partition coefficient, Henry’s law constant, volatility, phase transfer, environmental fate, in silico calculations

## Abstract

The temperature-dependent air–water partitioning behavior of a novel class of perfluoroalkyl and polyfluoroalkyl substances (PFAS) was assessed both experimentally and via *in silico* prediction. These PFAS contain ether or thioether linkages and are transformation products of an alternative PFAS surfactant. A modified version of the static headspace method with variable headspace/solution ratios was used to determine the dimensionless air/water partition coefficients (*K*_aw_) over a wide range of temperatures (25–80 °C). The samples were analyzed through the aqueous phase instead of the headspace because of their relatively low volatility. The obtained log *K*_aw_ values of the tested chemicals ranged from −2.6 to −1.0 at 25 °C. No differences in *K*_aw_ were observed between ether and thioether congeners with the same perfluorinated carbon chain length. Increasing the length of the perfluorinated carbon chain from CF_3_- to C_3_F_7_- increased *K*_aw_ by about 1.5 log units. The obtained *K*_aw_ of a well-studied fluorotelomer alcohol, 4:2 FTOH, matched those of previous studies, indicating the appropriateness of the method used. The temperature dependence of *K*_aw_, as quantified by the molar internal energy change of air–water partitioning, Δ*U*, ranged from 20 to 37 kJ/mol and was not substantially influenced by the structure of the chemicals. Among five *in silico* tools to predict air–water partitioning, the quantum chemistry-based COSMO*therm* ensured the most reliable and accurate prediction as compared to the experimental results.

## Graphical abstract

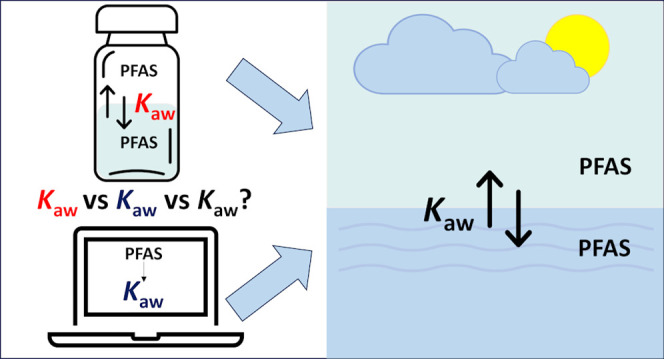

## 1 Introduction

Per- and polyfluoroalkyl substances (PFAS) are a group of industrial chemicals with a broad spectrum of physicochemical properties.^1^ The extensive usage, combined with persistence, and bioaccumulation or mobility of PFAS are responsible for their ubiquitous presence in the environment and biota.^2^ Moreover, PFAS have been linked to several human health concerns.^3^

Many of the regulated PFAS, such as perfluoroalkyl carboxylic acids and perfluoroalkyl sulfonic acids, contain a polar, ionizable functional group with a low acid dissociation constant (p*K*_a_). This causes them to be permanently negatively charged in water under environmentally relevant pH.^4^ However, some PFAS have a neutral (nonionized) functional group under environmentally relevant pH.^5^ These include fluorotelomer alcohols (FTOHs), perfluoroalkane sulfonamido ethanols (FASEs), fluorotelomer iodides (FTIs), fluorotelomer olefins (FTOs), and fluorotelomer (meth)­acrylates (FT­(M)­Acs), among others.^1^ Due to their lower water solubility, these neutral PFAS are expected to have higher air–water partition coefficients (i.e., Henry’s law constant) than predominantly ionic PFAS in water under environmental pH values. This will affect their environmental fate, which is illustrated by their detection in the atmosphere.^6,7^ Therefore, air–water partition behavior of neutral PFAS has been studied under laboratory settings.^8–13^

Many PFAS are persistent, while some can be degraded, leading to persistent, dead-end transformation products.^14^ For the neutral FTOHs, it has been shown that they can undergo transformation reactions by radicals in the atmosphere^15^ and by microbes under aerobic and anaerobic conditions^16^ to yield a series of persistent transformation products still fulfilling the PFAS definition.^17^

Due to the concerns related to PFAS, there has been an increasing interest in developing alternative PFAS that potentially mineralize or are benign in the environment. Comprehensive chemical safety assessment of substitutes and their transformation products is required to prevent regrettable substitutions in the future. This includes transport and fate assessment, which also requires the distribution of chemicals between mobile (water or air) and less mobile compartments (soil, sediment).

Within the “safe and sustainable by design” framework, the biodegradability of two prototype alternative fluorinated surfactants was tested in previous studies.^18,19^ These alternative PFAS (CF_3_–O-SURF and CF_3_–S-SURF, where SURF means surfactant) are surfactants made by functionalizing the structures CF_3_–O-ALC and CF_3_–S-ALC in Figure 1. Based on their structure, it was anticipated that the molecules were fully degradable, i.e., mineralizable, under environmental conditions; thus, avoiding the main concern regarding persistence. However, upon microbial transformation in activated sludge, these novel surfactants produced neutral fluorinated alcohol transformation products (TPs) that were predicted to be (semi)­volatile.^19^

**Figure 1:**
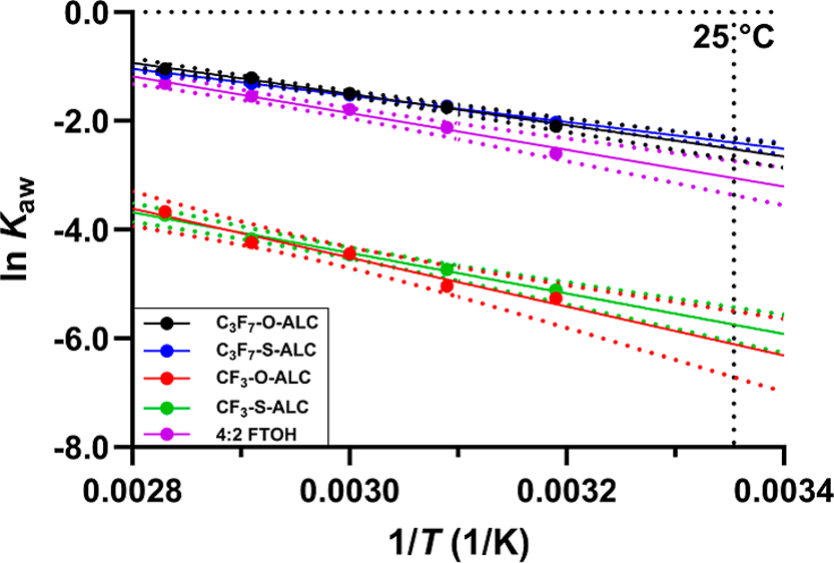
Experimental data are shown by presenting the mean of ln *K*_aw_ (dimensionless) values (data points) and their standard deviations (error bars) in terms of 1/*T* (K) for all the test chemicals, while the solid line indicates the result of the linear regressions according to eq 7. Please note that some error bars were too small to appear on the graph.

The aim of this study is therefore to investigate the volatility of these newly identified fluorinated alcohol TPs presented in Table 1 by studying their distribution between air and water and determining the air–water partition coefficient (*K*_aw_, dimensionless). For this purpose, a modified version of the static headspace method^20^ was used for the first time, where the depletion of the fluorinated alcohols in the aqueous phase was monitored in a test system with varying headspace/solution ratios at different temperatures. This method was validated by also testing a previously investigated FTOH (4:2 FTOH, Table 1) and comparing this *K*_aw_ value to those obtained by other methods from the literature. Moreover, the *K*_aw_ values were determined by various in silico models and compared to the experimental values.

**Table 1:**
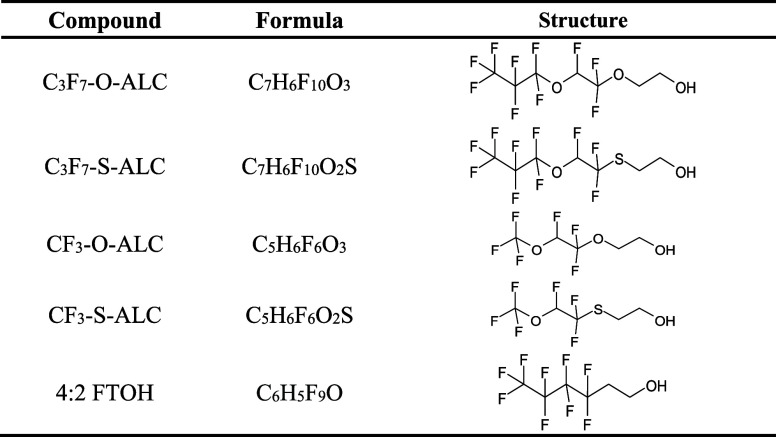
General Information About the Test Chemicals, Namely, the Transformation Products of a Novel Alternative Class of Fluorinated Surfactants CF_3_–O-SURF and CF_3_–S-SURF, as well as the Well-Studied Fluorinated Alcohol 4:2 FTOH

## 2 Theory

The classic static headspace method with varying headspace ratios was described earlier^17,21^ and implemented by many.^9,10,13,22^ An aqueous solution spiked with the chemical of interest is added to a series of vials with varying headspace/solution volume ratios. The chemical partitions into the headspace until equilibrium is reached. The different headspace/solution volume ratios result in different equilibrium concentrations.^20,21^ The relation between the headspace/solution ratio and the equilibrium concentration is used to derive the partition coefficient between water and air.

In the original method, only the air phase is sampled from the headspace and analyzed with a gas chromatograph (GC). The reciprocal GC peak areas are plotted against varying sample headspace/solution ratios. This results in a linear slope from which the dimensionless *K*_aw_ for the selected temperature can be derived.

In contrast to this previous work, in this study, the aqueous phase was sampled after equilibrium and analyzed using a liquid chromatograph (LC–MS). Reciprocal LC–MS peak areas were plotted against the different headspace/solution ratios to determine *K*_aw_. This relationship was concluded by using eqs 1 and 2.

1 $$K_{aw}=\frac{c_{hs}}{c_{sol}}$$

2 $$c_{0}=c_{sol}+c_{hs}\cdot \frac{V_{hs}}{V_{sol}}$$

The *c*_hs_ (mol·m^–3^) and *c*_sol_ (mol·m^–3^) are the equilibrium concentrations of the chemical of interest in headspace and solution, respectively. *V*_hs_ (m^3^) and *V*_sol_ (m^3^) are the volume of headspace and solution, respectively, while *c*_0_ (mol·m^–3^) is the initial concentration of the aqueous solution. Combining eqs 1 and 2 yields eq 3.

3 $$c_{0}=c_{sol}\left(1+K_{aw}\cdot \frac{V_{hs}}{V_{sol}}\right)$$

Dividing both sides of eq 2 by *c*_0_ and *c*_sol_ results in eq 3.

4 $$\frac{1}{c_{sol}}=\frac{1}{c_{0}}\left(1+K_{aw}\cdot \frac{V_{hs}}{V_{sol}}\right)$$

When assuming that *c*_sol_ is directly proportional to the intensity and area under the LC–MS signal, eq 4 can be represented as eq 5, where *RF* is the response factor in MS (*Area* = *RF*·*c*_sol_).

5 $$\frac{1}{\mathrm{area}}=\frac{1}{\mathrm{RF}\cdot c_{0}}\left(1+K_{aw}\cdot \frac{V_{hs}}{V_{sol}}\right)$$

Typically, 1/*area* (*y*) is plotted against *V*_hs_/*V*_sol_ (*x*), and then *K*_aw_ is expressed as the ratio of the slope (*K*_aw_/(*RF*·*c*_0_)) and the intercept (1/(*RF*·*c*_0_)). However, in this study, the nonlinear form of the equation (eq 6) was used based on the study of Hammer and Endo^23^ to easily provide the 95% confidence interval (CI) of *K*_aw_.

6 $$\mathrm{Area}=\frac{\mathrm{RF}\cdot c_{0}}{1+K_{aw}\cdot \frac{V_{hs}}{V_{sol}}}$$

To account for the temperature dependence of *K*_aw_, the internal energy change of air–water partitioning, Δ*U* (kJ·mol^–1^), was estimated using a modified Van’t Hoff-like equation

7 $$\ln K_{aw}=-\frac{\Delta U}{RT}+constant$$

where Δ*U* represents the molar internal energy change associated with transferring molecules from the water phase to the air phase, *T* is the absolute temperature (K), and *R* is the universal gas constant (8.3145···m^3^·Pa·K^–1^·mol^–1^). This approach assumes that Δ*U* is temperature-independent and that the system volume remains constant, as would be the case in a sealed and closed container.

To calculate the difference between the experimental and predicted log *K*_aw_ values, eq 8 was used.

8 $$\Delta \log K_{aw}=\log K_{aw}\;\left(experimental\right)-\log K_{aw}\;\left(predicted\right)$$

## 3 Materials and Methods

### 3.1 Standards and Chemicals

The novel fluorinated alcohols tested in this study, namely, CF_3_–O-ALC, CF_3_–S-ALC, C_3_F_7_–O-ALC, and C_3_F_7_–S-ALC (ALC stands for alcohol, Table 1, with SMILES presented in Table S1), were synthesized by Merck KGaA (Darmstadt, Germany). They are the TPs of prototype fluorinated surfactants, as was proven in previous studies.^18,19,24^ The 1*H*, 1*H*, 2*H*, and 2*H*-perfluorohexan-1-ol (4:2 FTOH, 97%) was purchased from Fluorochem UK (Hadfield, UK). We have no confirmed information about the purity of these chemicals by the producer. However, since this study uses relative changes for quantification, we assume that the exact purity does not impact our results.

Milli-Q water (Milli-Q Reference A+ system) was used throughout the experiments. Methanol (MeOH, ULC-MS grade) was acquired from Biosolve Chimie (Dieuze, France). Glacial acetic acid (≥99%) was obtained from Sigma-Aldrich (St. Louis, MO, USA).

### 3.2 Instrumental Analysis

All test chemicals (Table 1) were measured on an ultrahigh-performance liquid chromatograph coupled to a trapped ion mobility–time-of-flight mass spectrometer (UHPLC-timsTOF MS). Details of the instruments and the analytical method are presented in Text S1. Method performance was assessed with the linear range and instrumental limit of quantification (LOQ). This information, together with the expected *m*/*z* values and retention times of the test chemicals, is provided in Table S2.

### 3.3 Setup of the Air–Water Partition Experiment

Colorless borosilicate glass vials of 20 mL (VWR International BV, Amsterdam, Netherlands) with crimp caps and butyl septa (VWR International BV, Amsterdam, The Netherlands) were used throughout the whole experiment. The exact volume of the glass vials was determined by measuring the weights of the empty and fully filled vial with water at room temperature. The exact volume was calculated from the mass difference corrected for the water density at the given temperature. The mean volume of 20 vials was used in further calculations.

All test chemicals are liquids at room temperature. As carrier solvent, methanol was used to prepare individual stock solutions of 10 g/L. The mixed spiking solution containing the test chemicals in concentrations of 0.5 g/L for CF_3_–S-ALC and C_3_F_7_–S-ALC, and 1 g/L for CF_3_–O-ALC and C_3_F_7_–O-ALC and 0.5 g/L 4:2 FTOH, respectively, was prepared from the individual stock solutions. The aqueous test solution containing 400 μg/L CF_3_–O-ALC and C_3_F_7_–O-ALC, 200 μg/L CF_3_–S-ALC and C_3_F_7_–S-ALC, and 400 μg/L FTOH was freshly prepared prior to every experiment by adding 0.04 v/v % from the mix spiking solution to Milli-Q water in a polypropylene container. It was previously proved by others^13,22,25^ that a cosolvent of 0.03–0.1 v/v % had a negligible effect on the solubility of test chemical in the aqueous solution and thereby also on the *K*_aw_.

The vials were filled with 1, 2, 5, 10, 15, and 18 mL of the test solution in triplicate, resulting in the collection of vials with six different headspace/solution ratios. The test solutions were poured slowly to avoid aeration and, thus, loss of the chemicals. The vials were immediately closed with a butyl rubber septum and an aluminum seal. The triplicate series of the six headspace/solution collection was then equilibrated for 24 h at a constant temperature in the oven. In total, six different temperatures (25, 40, 50, 60, 70, and 80 °C) were tested.

Following this 24 h incubation, a short and a long needle were inserted through the septum ending in the headspace and bottom of the vial, respectively. Then, 0.75 mL from the test solution was withdrawn and directly mixed with 0.75 mL of MeOH in a 1.5 mL autosampler vial and stored in a freezer until analysis.

Applicability and reliability of this setup was assessed through the determination of adsorption loss and stability of the individual test chemicals. The details can be found in Text S2.

### 3.4 In Silico Prediction Models

To compare the experimental data from this study to model calculations for novel PFAS, five models—IFSQSAR from EAS-E Suite,^26^ UFZ-LSER,^27^ OPERA,^28^ HenryWin,^29^ and COSMO*therm* ^30^—were tested to calculate air–water partitioning. The UFZ-LSER model determines linear solvation energy relationship (LSER) compound and sorbing-phase descriptors and combines them for specific types of sorption-interactions (e.g., van der Waal, cavity formation, H-bonding) using LSER equations or other types of poly-parameter linear free energy relationships (PPLFERs). OPERA and HenryWin are quantitative structure-(activity) property relationships (QS­(A)­PRs) models based on model fragments; the IFSQSAR model predicts solute descriptors using a group contribution model, which can be further used to predict chemical partitioning properties by PPLFERs. COSMO*therm* differs from the other four models as it uses a combined quantum chemical-thermodynamical model. More details on the five models tested can be found in Text S3.

### 3.5 Data Handling

*K*_aw_ values under environmentally relevant conditions (25 °C) were determined both directly and indirectly.

In the direct method, *K*_aw_ and its 95% CI was determined by fitting the nonlinear eq 6. The nonlinear regression used a weighting factor of 1/*y*.^2^ For fitting, GraphPad Prism (version 10.1.2 for Windows, GraphPad Software, Boston, Massachusetts USA, www.graphpad.com) was used. If the relative standard deviation of the triplicates was larger than or equal to the relative deviation of the *area* values belonging to two vicinal headspace/solution ratios, those data were considered of low quality and were not used for further calculations.

The indirect method used the Van’t Hoff equation (eq 7) to plot the ln *K*_aw_ against the 1/*T* in Kelvin. Here, the *K*_aw_ values determined at temperatures of 40–80 °C were used to extrapolate the ln *K*_aw_ at 25 °C by linear regression. The linear regression was also performed by GraphPad Prism.

## 4 Results and Discussion

### 4.1 Experimental Results of Air–Water Partition Coefficients

The measured log *K*_aw_ values for the test compounds at different temperatures are presented in Table 2 and plotted against 1/*T* (1/K) in Figure 1. Extended information about the nonlinear regression analyses based on eq 6 at different temperatures of the test chemicals can be found in Table S3. It should be noted that the data do not perfectly follow eq 6, which is indicated by the coefficients of determination (*R* ^2^) being slightly lower than 1.00 for C_3_F_7_–O-ALC (*R* ^2^ from 0.976 to 0.999), C_3_F_7_–S-ALC (*R* ^2^ from 0.995 to 0.999), CF_3_–O-ALC (*R* ^2^ from 0.889 to 0.946), CF_3_–S-ALC (*R* ^2^ from 0.951 to 0.977), and 4:2 FTOH (*R* ^2^ from 0.966 to 0.997), with the weaker correlations tending to be at higher temperatures and for the smaller substances CF_3_–O-ALC and CF_3_–S-ALC. This could be due to potential interferences not accounted for in this study, particularly at higher temperatures. The log *K*_aw_ increased with temperature in the tested range for all the tested PFAS (Table 2 and Figures S2–S6). The *R* ^2^ obtained from the linear regressions between ln *K*_aw_ and 1/*T* (1/K), in eq 7, were >0.97 for all chemicals (Table 2). This indicates that the modified Van’t Hoff-like equation (eq 7) does well describe the internal energy change of air–water partitioning (Δ*U*) over this temperature range, implying that it is possible to interpolate partitioning to other temperatures within this temperature range.

**Table 2:** Dimensionless log *K*_aw_ Values (Mean [95% CI]) of Test Chemicals at Different Temperatures and Their Mean and Maximum Deviations from Each Other (Δ log *K*_aw_ Mean [Max])^a^

				log *K*_aw_ (dimensionless)						
	indirect				direct					
	slope (1/K)	*R* ^2^	Δ*U* (kJ·mol^–1^)	25 °C	25 °C	40 °C	50 °C	60 °C	70 °C	80 °C
C_3_F_7_–O-ALC	–2864 [−3312, −2416]	0.9928	23.8 [20.1, 27.5]	–1.09 [−1,17, −1,01]	–1.04 [−1.06, −1.01]	–0.91 [−0.92, −0.90]	–0.76 [−0.78, −0.74]	–0.65 [−0.68, −0.63]	–0.53 [−0.57, −0.49]	–0.45 [−0.54, −0.40]
C_3_F_7_–S-ALC	–2449 [−2669, −2229]	0.9976	20.4 [18.5, 22.2]	–1.04 [−1.08, −1.00]	–0.97 [−0.99, −0.95]	–0.88 [−0.90, −0.86]	–0.75 [−0.77, −0.73]	–0.66 [−0.68, −0.64]	–0.57 [−0.59, −0.55]	–0.49 [−0.53, −0.46]
CF_3_–O-ALC	–4502 [−6015, −2989]	0.9676	37.4 [24.9, 50.0]	–2.64 [−2.91, −2.38]	^a^	–2.29 [−2.40, −2.19]	–2.19 [−2.28, −2.11]	–1.93 [−2.07, −1.82]	–1.84 [−1.96, −1.75]	–1.59 [−1.74, −1.50]
CF_3_–S-ALC	–3729 [−4528, −2930]	0.9866	31.0 [24.4, 37.6]	–2.49 [−2.63, −2.35]	^a^	–2.22 [−2.27, −2.17]	–2.05 [−2.11, −2.00]	–1.94 [−2.01, −1.88]	–1.81 [−1.87, −1.76]	–1.62 [−1.70, −1.55]
4:2 FTOH	–3374 [−4115, −2634]	0.9859	28.1 [21.9, 34.2]	–1.32 [−1.45, −1.18]	–1.45 [−1.50, −1.40]	–1.13 [−1.20, −1.07]	–0.92 [−0.95, −0.90]	–0.78 [−0.85, −0.72]	–0.67 [−0.77, −0.59]	–0.57 [−0.67, −0.51]
Δlog *K*_aw_ (CF_3_–O-ALC vs CF_3_–S-ALC) mean [max]				0.15 [0.56]	^a^	0.07 [0.23]	0.14 [0.28]	0.01 [0.19]	0.03 [0.20]	0.03 [0.20]
Δlog *K*_aw_ (C_3_F_7_–O-ALC vs C_3_F_7_–S-ALC) mean [max]				0.05 [0.17]	0.07 [0.11]	0.03 [0.06]	0.01 [0.05]	0.01 [0.05]	0.04 [0.10]	0.04 [0.13]
Δlog *K*_aw_ (CF_3_–O-ALC vs C_3_F_7_–O-ALC) mean [max]				1.55 [1.90]	^a^	1.38 [1.50]	1.43 [1.54]	1.28 [1.44]	1.31 [1.47]	1.14 [1.34]
Δlog *K*_aw_ (CF_3_–S-ALC vs C_3_F_7_–S-ALC) mean [max]				1.45 [1.63]	^a^	1.34 [1.41]	1.30 [1.38]	1.28 [1.37]	1.24 [1.32]	1.13 [1.24]

a Slopes (mean [95% CI]) (1/K) and the coefficients of determination (*R* ^2^) of the regression lines of ln *K*_aw_ (dimensionless) vs 1/*T* (1/K) (eq 5), as well as the Δ*U* (mean [95% CI]) (kJ·mol^–1^) values (eq 7).

b Not applicable.

In the case of 4:2 FTOH, C_3_F_7_–O-ALC, and C_3_F_7_–S-ALC, the differences in the log *K*_aw_ values at 25 °C determined by the direct or indirect approach were negligible (Table 2). The log *K*_aw_ of CF_3_–O-ALC and CF_3_–S-ALC was too low to be directly determined at 25 °C; however, due to the higher volatility at higher temperatures (40–80 °C), the indirect method enabled for extrapolation to 25 °C according to eq 8. The *K*_aw_ values of CF_3_–O-ALC and CF_3_–S-ALC at 25 °C were extrapolated from higher temperatures, with the accuracy of this extrapolation presented in Table 2.

By comparison of the log *K*_aw_ values of the fluorinated alcohols, a maximum difference of 0.56 between CF_3_–O-ALC and CF_3_–S-ALC or 0.17 between C_3_F_7_–O-ALC and C_3_F_7_–S-ALC was observed (Table 2). The length of the fluorocarbon chain −CF_3_– vs C_3_F_7_–, however, caused a mean of 1.3 log units of difference in the *K*_aw_ over the whole temperature range (Table 2). The *K*_aw_ values of C_3_F_7_–O-ALC and C_3_F_7_–S-ALC were similar to the *K*_aw_ of 4:2 FTOH (Table 2 and Figure 1). Plots used for the determination of *K*_aw_ by the direct and indirect approach are presented on Figures 1 and S2–S6.

This is the first study reporting on air–water partition coefficients of CF_3_–O-ALC, CF_3_–S-ALC, C_3_F_7_–O-ALC, and C_3_F_7_–S-ALC, and therefore those results cannot be compared to literature data. However, volatility of the structurally similar 4:2 FTOH was investigated in different studies previously,^9,10,12,13^ of which data are compared to current results in Table 3. Our experimentally determined log *K*_aw_ values at 25 °C—–1.45 [95% confidence interval (CI): −1.50, −1.40] and −1.32 [95% CI: −1.45, −1.18] by the direct and indirect method, respectively—were similar to those of Goss et al.^10^ (−1.52) and Endo et al.,^9^ who used the classic static headspace (−1.56) and the thermodynamic cycle method (−1.57), and similar within 0.2–0.3 log units with the result of Wu and Chang^12^ (−1.21), who used the integrated gas-stripping method. However, the log *K*_aw_ values determined by Lei et al.^11^ and Abusallout et al.^13^ differed from our study and those of the other three studies (Table 3). It was described by others^10^ that the experimental values and trends for FTOHs by Lei et al. are erroneous because they did not account for sorption artifacts with the glass walls and lid, therefore it was dismissed hereinafter. Endo et al.^9^ attributed the deviation of their *K*_aw_ values from those of Abusallout et al.^13^ to either vial sorption or air–water interface interactions. The vial sorption can likely be excluded since Abusallout et al.^13^ gave similar results in experiments conducted at two concentration levels. However, this observation is not in line with other studies that showed significant adsorption of longer FTOHs to glass surfaces.^10,12^ Regarding the air–water interfacial effects, Endo et al.^9^ used the hexadecane/air/water thermodynamic cycle method to intentionally minimize air–water interfacial effects when determining *K*_aw_. Meanwhile, no such effort was made by any other studies—including the current one and the one of Abusallout et al.^13^ We therefore cannot provide a clear explanation for the discrepancies of air–water partitioning of 4:2 FTOH between Abusallout et al.^13^ and the other studies.

**Table 3:** Comparison of the Experimentally Determined Dimensionless log *K*_aw_ (Mean [95% CI]) Data from This Study to Experimental Literature Data at 25 °C for 4:2 FTOH

reference	log *K*_aw_ (dimensionless)
Lei et al.^11^ ^,^ ^a^	1.83
Goss et al.^10^ ^,^ ^a^	–1.52
Wu and Chang^12^ ^,^ ^b^	–1.21 [−1.30, −1.12]
Abusallout et al.^13^ ^,^ ^a^	–0.51 [−0.56, −0.45]
Endo et al.,^9^ direct^a^	–1.56
Endo et al.,^9^ indirect^c^	–1.57
this study, direct	–1.45 [−1.50, −1.40]
this study, indirect	–1.32 [−1.45, −1.18]

a The following experimental methods were used: classic static headspace method with variable headspace/solution ratios.

b Integrated gas-stripping method, using stainless steel vessels.

c Determined using the equation *K*_aw_ = *K*_Hxd/w_/*K*_Hxd/air_ which is based on the thermodynamic cycle method.

We wanted to strengthen the reliability of our method by comparing *K*_aw_ data for 6:2 and 8:2 FTOH as well. However, experiments with 6:2 and 8:2 FTOH showed losses of 31 ± 9% and 59 ± 9%, respectively, on the wall/lid surfaces and interfaces. These losses would have led to the determination of erroneous *K*_aw_. More details on this experiment can be found in Text S2. Similarly, Goss et al.^10^ stated that their calculated *K*_aw_ value for 8:2 FTOH must be incorrect because presumably 50–80% of the total mass of 8:2 FTOH sorbed to the interfaces when using glass vials. Wu and Chang^12^ also reported the increased adsorption and thereof unreliable *K*_aw_ values of 8:2 FTOH in glass vials. However, in contrast to this study, they did not observe significant adsorption for 6:2 FTOH, which might be explained by the different materials and dimensions of the glass tube.^10,12^

The impact of adsorption on *K*_aw_ determination by the static headspace or gas-stripping method is also reported in the literature. While the calculated *K*_aw_ values of 4:2 FTOH were mostly in accordance with each other (Table 3), the calculated *K*_aw_ values for 6:2 FTOH: −0.56,^10^ 0.37,^12^ 0 × 10,^13^ −0.64 ^9^ and 8:2 FTOH: −1.20,^10^ 0.31,^12^ 0.30,^13^ 0.11 ^9^ showed more deviation in the literature. Here, it should be emphasized again that the reported values for 6:2 and 8:2 FTOH from the study of Endo et al.^9^ were determined by the thermodynamic cycle method to minimize third-phase sorption, while other studies used methods that can easily be biased by unique artifacts depending on the method, such as water surface sorption artifacts increasing the *K*_aw_ becoming more pronounced for experiments using the gas-stripping method.^12,31^

Overall, we concluded the usability of our method for substances with low adsorption potential, sufficient water solubility, and relatively low volatility based on the agreement of our experimental *K*_aw_ values of 4:2 FTOH. This implies the method applied here is suitable to derive *K*_aw_ values for structurally similar novel fluorinated alcohols. Additional considerations would be needed for larger PFAS.

### 4.2 In Silico Results of Air–Water Partition Coefficients

Experimental log *K*_aw_ values were compared to computationally estimated values (Table 4). For 4:2 FTOH, IFSQSAR predicted log *K*_aw_ values identical to our experimental values. The prediction of the UFZ-LSER tool was also not substantially different from our experimental log *K*_aw_. The predictions for HenryWin (Δlog *K*_aw_ = ∼−0.7) and COSMO*therm* (Δlog *K*_aw_ = ∼0.6) were well within an order of magnitude; however, the prediction by OPERA largely differed and covered 3 orders of magnitude (Table 4).

**Table 4:** Comparison of the Experimentally Determined Dimensionless log *K*_aw_ (Mean [95% CI] Values of This Study to the log *K*_aw_ (±error) of the In Silico Model Predictions at 25 °C^a^

	experimental values		estimated values				
	this study, direct	this study, indirect	IFSQSAR	UFZ-LSER	OPERA	HenryWin	COSMO*therm*
	log *K*_aw_ mean [95% CI]	log *K*_aw_ mean [95% CI]	log *K*_aw_ ± error	log *K*_aw_	log *K*_aw_ ± error	log *K*_aw_	log *K*_aw_
C_3_F_7_–O-ALC	–1.04 [–1.06, −1.01]	–1.09 [–1.17, −1.01]	*–1.61* ± *1.88*	*–3.17*	*–4.16 (+1.66; −1.09)*	–4.34	–0.99
C_3_F_7_–S-ALC	–0.97 [–0.99, −0.95]	–1.04 [–1.08, −1.00]	*–3.24* ± *2.10*	*–4.43*	*–4.16 (+1.66; −1.09)*	–4.38	–1.77
CF_3_–O-ALC	N/A	–2.64 [–2.91, −2.38]	–3.11 ± 1.50	*–4.64*	*–5.53 ± 2.85*	–5.78	–2.42
CF_3_–S-ALC	N/A	–2.49 [–2.63, −2.35]	*–4.75* ± *1.78*	*–5.89*	*–4.34 ± 1.51*	–5.82	–2.85
4:2 FTOH	–1.45 [–1.50, −1.40]	–1.32 [–1.45, −1.18]	*–1.38* ± *0.34*	*–1.54*	*–3.95 (+1.51; −0.96)*	–0.65	–1.99^b^

a Please note that the values presented in italic were outside of the model’s application domain. N/A = not available.

b Adapted from Endo et al.^9^

UFZ-LSER (∼2 < Δlog *K*_aw_ < ∼3), OPERA, and HenryWin (Δlog *K*_aw_ ∼ 3) largely underpredicted the volatility of the novel fluorinated alcohols (Table 4). HenryWin gave a very consistent underprediction (Δlog *K*_aw_ ∼ 3) for all the four novel chemicals compared to the experimental data, while the Δlog *K*_aw_ for the ether congeners were 1 order of magnitude smaller than those for the thioether congeners—∼2 vs ∼3, respectively—in the case of UFZ-LSER (Table 4). It should be noted that the predicted solute descriptors by UFZ-LSER and (some of) the log *K*_aw_ values by OPERA and IFSQSAR were outside the application domain of the tools, indicating that predictions are unreliable (Table 4). Also, IFSQSAR and OPERA provided predictions with high uncertainties.

The log *K*_aw_ values of the ether congeners predicted by COSMO*therm* had no or negligible difference from the experimentally determined values for CF_3_–O-ALC and C_3_F_7_–O-ALC, respectively. However, COSMO*therm* predicted a substantially lower volatility for the thioether than ether congeners—log *K*_aw_ = −0.99 vs −1.77 in the case of the C_3_F_7_-compounds and log *K*_aw_ = −2.42 vs −2.85 in the case of the CF_3_-compounds, respectively—while the experimental values for the C_3_F_7_-and CF_3_-compounds showed no differences between them (Table 4). Overall, compared to our experimental results, COSMO*therm* provided the most accurate predictions of all of the in silico methods. Our experience regarding the accuracy of the various in silico predictions is in line with the observation of Endo et al.,^9^ in which large prediction errors of the HenryWin bond contribution method (same version as in this study) were reported for several neutral PFAS. In their case, however, this model tends to overestimate *K*_aw_. OPERA calculations were also not trustworthy in their case as the local application domain indices were outside of the global application domain (<0.4). Similar to this study, COSMO*therm* ensured the highest accuracy.^9^ Arp et al. also found that COSMO*therm* made predictions usually within 1 order of magnitude of the experimental value, while HenryWin performed more inaccurately.^8^

The overall low performance of the four QSAR-and LSER-based methods is not surprising, as they are calibrated and thereby highly dependent on the size, diversity, and quality of the data set.^32^ Experimental data on physicochemical properties of PFAS are still scarce and fraught with high discrepancies. Overall, these results suggest that the QSAR- and LSER-based models require more and better training data sets in order to provide more accurate predictions in the future for novel classes of volatile PFAS. Obtaining reliable partitioning data does not only require considerable effort to build representative training sets but also suffers from practical issues as surfactant-like properties of many PFAS that hamper partitioning experimentation.^33^

### 4.3 Structural Patterns in the Air–Water Partition Coefficients and Internal Energies

The CF_3_–O-ALC and C_3_F_7_–O-ALC, as well as CF_3_–S-ALC and C_3_F_7_–S-ALC, are homologous chemicals as they differ in –C_2_F_4_-moieties from each other. As indicated in Table 2, the addition of the C_2_F_4_-moiety increased the log *K*_aw_ value (∼1.5 log units at 25 °C) in our study. Goss et al.^10^ reported a 0.96 log unit difference in log *K*_aw_, when comparing 4:2 FTOH to 6:2 FTOH. Endo et al.^9^ determined a 0.93 log unit difference between 4:2 FTOH and 6:2 FTOH and a 0.75 log units difference between 6:2 FTOH and 8:2 FTOH. Endo et al.^9^ determined the effect of CF_2_-chain length by comparing data of two *X*:1 FTOHs, three *X*:2 FTOHs, three unsubstituted perfluoroalkane sulfonamides, and two *N*-methyl perfluoroalkane sulfonamides. Log *K*_aw_ of these four groups increased consistently by 0.43 ± 0.02 log units per CF_2_ unit (i.e., by 0.86 log units per –C_2_F_4_-unit). The current study reports a greater increase in log *K*_aw_ per –C_2_F_4_-units than the studies listed above. The log *K*_aw_ values are dependent on the intermolecular interactions with the surrounding phases, including polar (e.g., hydrogen bonding) and nonpolar (i.e., van der Waals) interactions. The –CF_2_-unit has two effects on the molecule: (i) increasing hydrophobicity (increasing size and cavity formation energy costs with limited increase in van der Waals interactions) and (ii) exerting an electron-withdrawing effect on the neighboring polar functional group.^34^ The polyfluoroalkyl chain of the studied fluorinated alcohols is directly connected to the ether O atom, hampering the H-bond capacity of the O atom and decreasing the water solubility (i.e., increases log *K*_aw_). The electron-withdrawing effect is expected to be more substantial in the C_3_F_7_- than in CF_3_-compounds, causing an increased hydrophobicity and higher log *K*_aw_ with increasing perfluorinated chain length thereof. Therefore, the hydrophobicity increment due to the addition of the –C_2_F_4_-unit accounts for ∼0.9 log unit increase of log *K*_aw_, while the additional 0.6 log unit increase of log *K*_aw_ could be due to the decreased H-bond donating property of the ether and thioether functional groups. The previous studies were not able to find such a difference, as they compared PFAS with a perfluorinated chain of C_4_F_9_– and longer, which could be too separated from the polar functional group to show a substantial difference in the electron-withdrawing effect.

The *K*_aw_ values determined at different temperatures enabled us to describe the dependence of air–water transition on temperature, namely, calculating the molar internal energy change of water–air partitioning (Δ*U*) based on the modified Van’t Hoff-like equation (eq 7 and Table 2). Despite the unequivocal incremental effect of the addition of the –CF_2_-unit on log *K*_aw_, we could not conclude the effect of molecular size on Δ*U*, as the difference of Δ*U* between the ether congeners was smaller than detectable due to the relatively large error. The Δ*U* was 37.4 [24.9, 50.0] and 23.8 [20.1, 27.5] for CF_3_–O-ALC and C_3_F_7_–O-ALC, respectively. While a slight decrease in the Δ*U* of the thioether congeners—31.0 [24.4, 37.6] vs 20.4 [18.5, 22.2] kJ·mol^–1^ for CF_3_–S-ALC and C_3_F_7_–S-ALC, respectively—was observed (Table 2). This implies that the increasing molecular size either did not affect or slightly decreased the temperature dependence of air–water partitioning.

### 4.4 Application Domain of the Static Headspace Method Used in This Study

The current setup was suitable to determine the air–water partition coefficients of fluorinated alcohols possessing at least two but less than six fully fluorinated carbon atoms. Using this method, we obtained *K*_aw_ data over 1.6 log units from about −2.6 to −1.0. Endo et al. achieved reliable log *K*_aw_ values over a wider range from ca. −2.0 to −0.1, by the headspace method.^9^ The air–water partitioning of compounds that are less volatile, with a log *K*_aw_ below −2.6, would require more advanced analytics than those in the current setup. Similarly, chemicals with high adsorption coefficients require alternative methods for the determination of *K*_aw_, e.g., as described by Endo et al.^9^

### 4.5 Environmental Implications

This modified version of the static headspace method is suitable for determining the log *K*_aw_ of many neutral chemicals, which fall within the above-mentioned application domain. The obtained experimental data showed that current LSER/QSAR models were not able to predict the air–water partitioning as accurately as the COSMO*therm* model. Experimental data, such as obtained in this study, are needed to improve these models, which enable them to better assess the environmental fate of PFAS including transformation products of alternative PFAS that might be introduced to the market.

Even though there are no official regulatory criteria set for the determination of the Henry’s Law constant, some indicative values were provided in the OECD (Organisation for Economic Co-operation and Development) 309 guideline.^35^ Namely, compounds with log *K*_aw_ < −3.39 can be regarded as nonvolatile, with −3.39 < log *K*_aw_ ← 1.39 as semivolatile and with −1.39 < log *K*_aw_ as volatile in practice. The novel fluorinated alcohols CF_3_–O-ALC and CF_3_–S-ALC would be considered semivolatile, while C_3_F_7_–O-ALC and C_3_F_7_–S-ALC are considered volatile, accordingly. A more detailed environmental fate assessment in terms of the log *K*_aw_ values could be provided by knowing the corresponding octanol–water partition coefficients (*K*_ow_).

In previous studies, the atmospheric fate and reactivity of the four novel fluorinated alcohols addressed in this study were investigated^36,37^ (see Text S4). Those findings demonstrate the importance of understanding the environmental behavior of both the parent substances and their transformation products. Only then can we provide a comprehensive and reliable basis for environmental fate and impact assessments.

## Supplementary Material



## Notes

Detailed description of instrumental analysis; information about the adsorption and stability of test chemicals; detailed description of in silico prediction models; short review of the reactivities of the addressed chemicals; SMILES strings of the test and reference chemicals; additional information to the LC-timsTOF MS data evaluation and method performance; information about the nonlinear regression analyses according to eq 6 at different temperatures of the test chemicals; proposed transformation pathway of the alternative fluorinated chemicals addressed in this study; plots used to the determination of *K*_aw_ for the test chemicals by direct approach; and linearity range of the test chemicals (PDF)
