# Supplementary material for: Determining the Temperature-Dependent Air–Water Partitioning of Ether- and Thioether-Alcohol Perfluoroalkyl and Polyfluoroalkyl Substances Using a Modified Static Headspace Method
Source: Environ Sci Technol. 2025 Jul 28;59(31):16513–20. doi: 10.1021/acs.est.4c11447 (PMC12355946; doi:10.1021/acs.est.4c11447)
Supplement: Supplementary file 1 [file es4c11447_si_001.pdf]

Supporting Information

Determining the temperature-dependent air-water partitioning of ether- and thioether-alcohol PFAS using a modified static headspace method

Viktória Licul-Kucera[1], Annemarie P. van Wezel[1], Hans Peter H. Arp,[2,3], Thomas L. ter Laak[1,4]*

[1]Institute for Biodiversity and Ecosystem Dynamics, University of Amsterdam, Science Park 904, 1098 XH Amsterdam, The Netherlands

[2]Norwegian Geotechnical Institute, P.O. Box. 3930, Ullevål Stadion, N-0806 Oslo, Norway

[3]Norwegian University of Science and Technology, 7024 Trondheim, Norway

[4]KWR Water Research Institute, P.O. Box 1072, 3430 BB Nieuwegein, The Netherlands

*Corresponding author: v.liculkucera@uva.nl or licul.k.viktoria@outlook.hu

Supporting Information contains:

- Four text chapters
- Three tables
- Eleven figures

Table of contents

Name	Page
Text S1: Instrumental analysis	S4-5
Text S2: QA/QC	S6-7
Text S3: *In-silico* prediction models	S8-9
Text S4: Short review of the reactivities of the addressed chemicals	S10
Table S1: SMILES strings of the test and reference chemicals	S11
Table S2: Additional information to the LC-timsTOF MS data evaluation and method performance	S12
Table S3: Information about the nonlinear regression analyses according to Eq. 6 at different temperatures of the test chemicals.	S13
Fig. S1: Proposed transformation pathway of the alternative fluorinated chemicals addressed in this study	S14
Fig. S2: Plots used to the determination of K_{aw} for C_3F_7-O-ALC by direct approach	S15
Fig. S3: Plots used to the determination of K_{aw} for C_3F_7-S-ALC by direct approach	S16
Fig. S4: Plots used to the determination of K_{aw} for CF_3-O-ALC by direct approach	S17
Fig. S5: Plots used to the determination of K_{aw} for CF_3-S-ALC by direct approach	S18
Fig. S6: Plots used to the determination of K_{aw} for 4:2 FTOH by direct approach	S19
Fig. S7: Linearity range of C_3F_7-O-ALC.	S20
Fig. S8: Linearity range of C_3F_7-S-ALC.	S21
Fig. S9: Linearity range of CF_3-O-ALC.	S22
Fig. S10: Linearity range of CF_3-S-ALC.	S23

Fig. S11: Linearity range of 4:2 FTOH. S24

References S25-27

Text S1: Instrumental analysis

The Nexera XS UHPLC system with controller, pumps, degassing unit, autosampler and oven was from Shimadzu ('s-Hertogenbosch, Netherlands), while the timsTOF Pro 2 MS was a from Bruker (Leiderdorp, Netherlands). The compounds were separated on an Acquity UPLC CSH C18 column (130 Å, 2.1 × 150 mm, 1.7 µm). The mobile phase consisted of 0.05 % acetic acid in water (A) and MeOH (B). The gradient started at 20% eluent B, increased to 100% B in 6 min, and held it for 4 min. Then eluent B decreased to 20% from 10-10.5 min and the column was re-equilibrated with the starting composition for 2 min. The oven temperature was set on 40 °C. The injection volume was 20 µL. The ion polarity for all recorded MS scans was negative. Vacuum Insulated Probe Heated Electrospray Ionization (VIP-HESI) source was used, with a capillary voltage of 4500 V and end plate offset of 500 V. The nebulizer gas set was 2.0 bar, the dry gas flow was 8.0 L/min, and the dry gas temperature was 230 °C. The sheath gas flow was 4.0 L/min, the sheath gas temperature was 150 °C. The scans were recorded in the m/z range=20-1300. The trapped ion mobility function was off throughout the whole measurement. TASQ software (Bruker Daltonics GmbH&Co, version number: 2023.1.5 10496) was used for data analysis.

Neutral PFAS such as fluorotelomer alcohols (FTOHs) are normally not determinable as molecular ions $[M-H]^-$ by ESI, but they can be identified and also quantified as the acetate adduct of the molecular ions $[M+CH_3COO]^-$, where acetate ions originate from the mobile phase [1–3].

As LC areas are used in the calculation, no calibration curves for quantification were used. Determining and working in the linear concentration-area range of the compounds is however a requirement of this approach. Therefore, the linear range of each individual test and reference chemicals was determined by injecting standards in

the 0.01-400 μg/L range. Linearity in the selected range was accepted if the goodness of the fitted linear (with a weighting factor of $1/x^2$) (R^2) > 0.995. In order to verify the accuracy and precision of the method, a standard of 100 μg/L was injected regularly, and the recovery was evaluated.

Text S2: QA/QC

Adsorption loss

The 1H, 1H, 2H, 2H-perfluorooctan-1-ol (6:2 FTOH, 98%) was obtained from ABCR (Karlsruhe, Germany), and 1H, 1H, 2H, 2H-perfluorodecan-1-ol (8:2 FTOH, 97%) was bought from Sigma-Adrich (St. Louis, MO, USA).

Applicability of the setup used in this study was first evaluated through the determination of the adsorption of the chemical to a third phase, e.g., wall of the glass tube or the lid. The adsorption loss was determined by comparing the LC-MS response of the freshly prepared test solution aliquot (*n*=1) to that of the test solution aliquots (*n*=2) which completely, headspace-free topped the vials and were equilibrated at selected temperatures (25, 40, 50, 60 °C) for 24 hours. Even though adsorption is normally temperature-dependent [4], no significant correlation between the adsorption loss and temperature was observed in this study, probably due to the negligible or small adsorption losses. Therefore, the adsorption loss of a compound was calculated as the average of the individual replicate values determined at different temperatures according to Eq. S1. The uncertainty for adsorption loss was expressed as standard deviation of all samples (*n*=8).

$$Adsorption\ loss\ (\%) = \frac{Area_{\text{fresh test solution}} - Area_{\text{full vial}}}{Area_{\text{fresh test solution}}} \times 100\% \qquad \text{Eq. S1}$$

The longer fluorocarbon chain, the higher was the adsorption to surfaces. Almost negligible adsorption factors – 0±1% and 4±6% – were reported for the CF_3-S-ALC and CF_3-O-ALC, respectively, with the lowest amount (three) of fluorinated carbon atoms each. The 4:2 FTOH, C_3F_7-O-ALC and C_3F_7-S-ALC with four, five and five fluorinated carbon atoms had an adsorption factor of -8±6%, 11±2% and 14±4%, respectively. These relatively low adsorption losses were not corrected for, also because the adsorption between wall and water, wall and air and the water or air and the lid is

not fully known. Therefore, it was assumed that the marginal adsorption of the fully filled vials had marginal impact on the obtained K_{aw}. Besides 4:2 FTOH, the longer-chained homologues 6:2 and 8:2 FTOH were also tested for adsorption (initial concentration in the test solution was 400 µg/L for each) and had an adsorption loss of 31±9% and 59±9%, respectively. These adsorption losses were considered as non-negligibly high which would presumably have led to erroneous K_{aw} calculation as seen in other studies. Therefore the 6:2 and 8:2 FTOH could not be further analyzed.

Stability of the test chemicals

In order to ensure the reliability of the study, a possible spontaneous or heat-activated transformation or degradation of the test chemicals was also investigated. For this, samples were checked for expected TPs such as the first oxidation products – carboxylic acids – of the fluorinated alcohols as well their TP – also a carboxylic acid – which can be produced by the consecutive elimination of a HF molecule(s) [3,5]. This theoretical transformation pathway of the test chemicals can be seen on Fig. S1.

In all test samples at 70 and 80 °C, a TP at 3.0 min with m/z about 160.9867 was detected. It was identified as $C_3H_2F_4O_3$, the dead-end TP of CF_3-O-ALC and CF_3-S-ALC, previously described in Licul-Kucera et al [3]. This product was also confirmed with a reference standard. The concentration of this product was 2.0 ± 0.2 and <0.5 ng/mL at 80 °C and 70 °C, respectively. Assuming the complete transformation of CF_3-O-ALC and CF_3-S-ALC prior to the partition to the headspace, these concentrations would correspond to only 0.5% and 0.2% of the total theoretical produced amount. Consequently, we assumed that the formation of this transformation product did not bias the determined K_{aw} values.

Text S3: *In-silico* prediction models

IFSQSAR version 1.1.1 by EAS-E Suite [6] is based on Iterative Fragment Selection – Quantity Structure Activity Relationships (IFS-QSARs), which iteratively generates and selects fragments that can be used to predict a physical-chemical property based on multiple linear regression [7]. This model also derives Abraham solvation parameters, which themselves can be correlated to physical chemical properties through Linear Solvation Energy Relationship (LSER), similar to the UFZ-LSER model [8].

The UFZ-LSER model [9] is also built on a similar combination of IFS-QSARs and LSERs. These databases contain experimentally determined solute descriptors for the reference compounds addressed in this study, while for the test chemicals descriptors are predicted based on the calibration using existing experimental data for these and other substances. This means that the reliability of solute predictions for chemicals where no experimental data exists is strongly dependent on the training dataset [10]. The model indicates if predictions fall with thin the application domain – which should be always considered before relying on the predicted value.

OPERA (Open (Quantitative) Structure-activity/property Relationship App, version 2.9.1, www.eas-e-suite.com. Accessed 22-01-2024.) which predicts the physicochemical properties based on a weighted k-nearest neighbor approach with a minimum number of required descriptors [11], was accessed through EAS-E Suite.

HenryWin (version 3.21) is a module of EPI Suite (US EPA. Estimation Programs Interface Suite™ for Microsoft® Windows, v 4.11. Accessed 22-01-2024. United States Environmental Protection Agency, Washington, DC, USA), a physicochemical property and environmental fate estimation program. It uses the bond and group contribution methods originally described by Hine and Mookerjee [12]; however, for diverse PFAS generally the bond method (based on chemical bonds

present) provides output, as the group method only works for groups defined within the application domain [13].

Unlike the abovementioned QSAR/QSPR-based methods, COSMO*therm* calculations do not require training sets for the prediction of physicochemical properties as they are based on the Conductor-like Screening Model for Real Solvents (COSMO-RS) theory [14,15]. Namely, the results of quantum chemical calculations are used based on statistical thermodynamics to predict thermodynamic equilibrium properties such as partition coefficients of fluids and liquid mixtures. COSMO*therm* calculations are performed by the software called COSMO*conf*, TURBOMOLE (version 7.2) and COSMO*therm* (version 2020) (all BIOVIA, Dassault Systèmes, San Diego, USA). First, an initial structure of the molecule as SMILES string is entered into COSMO*conf* which then identifies the different possible conformations of the molecule and evaluates them based on their relative energy. For these quantum chemical calculations, COSMO*conf* uses the TURBOMOLE program package. COSMO files are then generated which can be used as input to COSMO*therm*. Different parametrizations are possible in COSMO*conf*, here we used the BP-TZVPD-FINE parametrization.

Text S4: Short review of the reactivities of the addressed chemicals

The presence of the thioether group resulted in a 2.4 or 2.9-times increase in the gas-phase OH radical reactivity over the ether congeners for the C_3F_7- and CF_3-alcohols, respectively[16]. The gas-phase rate constants of the two thioether congeners was way much faster, that of the C_3F_7-O-ALC was similar, while that of the CF_3-O-ALC was slower than that of the n:2 FTOHs (n=4,6,8)[16,17]. The C_3F_7-S-ALC had the shortest atmospheric lifetime of all the four fluorinated alcohols, while only CF_3-S-ALC demonstrated the potential for complete mineralization[16]. In the gas phase, simple species such as CO, CO_2, SO_2, COF_2, CHFO were formed[16,18]. In the aqueous phase, moreover, the persistent and mobile perfluoropropionic acid (PFPrA) and other short polyfluoroether acid species as terminal products from the C_3F_7-compounds were formed[16,18].

Table S1: SMILES strings of the test and reference chemicals.

Compound	Canonical SMILES
C_3F_7-O-ALC	OCCOC(C(OC(C(C(F)(F)F)(F)F)(F)F)F)(F)F
C_3F_7-S-ALC	OCCSC(C(OC(C(C(F)(F)F)(F)F)(F)F)F)(F)F
CF_3-O-ALC	OCCOC(C(OC(F)(F)F)F)(F)F
CF_3-S-ALC	OCCSC(C(OC(F)(F)F)F)(F)F
4:2 FTOH	OCCC(C(C(C(F)(F)F)(F)F)(F)F)(F)F
6:2 FTOH	OCCC(C(C(C(C(C(F)(F)F)(F)F)(F)F)(F)F)(F)F)(F)F
8:2 FTOH	OCCC(C(C(C(C(C(C(C(F)(F)F)(F)F)(F)F)(F)F)(F)F)(F)F)(F)F)(F)F

Table S2: Additional information to the LC-timsTOF MS data evaluation and method performance.

	**Theoretical m/z of $[M+CH_3COO]^-$**	**Retention time (min)**	**Linear range (ng/mL)**	**Instrumental LOQ (ng/mL)**
C_3F_7-O-ALC	387.0296	5.8	0.1-200	0.1
C_3F_7-S-ALC	403.0067	6.0	0.01-100	0.01
CF_3-O-ALC	287.0360	4.7	0.5-200	0.5
CF_3-S-ALC	303.0131	5.1	0.05-100	0.05
4:2 FTOH	323.0335	5.7	10-200	10
6:2 FTOH	423.0271	6.4	0.1-200	0.1
8:2 FTOH	523.0207	6.8	0.5-200	0.5

Abbreviations: iLOQ=instrumental limit of quantification, equals to the corresponding lowest point of the linear range.

Table S3: Information about the nonlinear regression analyses according to Eq. 6 at different temperatures of the test chemicals.

	C_3F_7-O-ALC			C_3F_7-S-ALC			CF_3-O-ALC			CF_3-S-ALC			4:2 FTOH		
	K_{aw} mean [95%CI]	R^2	Sx.z	K_{aw} mean [95%CI]	R^2	Sx.z	K_{aw} mean [95%CI]	R^2	Sx.z	K_{aw} mean [95%CI]	R^2	Sx.z	K_{aw} mean [95%CI]	R^2	Sx.z
**80 °C**	0.3528 [0.2882; 0.4019]	0.9755	0.1042	0.3257 [0.2981; 0.3498]	0.9945	0.0495	0.0255 [0.0183; 0.0317]	0.8909	0.0587	0.0240 [0.0198; 0.0279]	0.9513	0.0361	0.2689 [0.2154; 0.3098]	0.9686	0.1111
**70 °C**	0.2983 [0.2661; 0.3253]	0.9909	0.0619	0.2693 [0.2544; 0.2834]	0.9974	0.0326	0.0144 [0.0109; 0.0179]	0.9164	0.0308	0.0154 [0.0134; 0.0175]	0.9746	0.0175	0.2149 [0.1690; 0.2559]	0.9657	0.1053
**60 °C**	0.2245 [0.2112; 0.2363]	0.9971	0.0330	0.2178 [0.2081; 0.2272]	0.9983	0.0254	0.0118 [0.0085; 0.0150]	0.8888	0.0302	0.0116 [0.0098; 0.0133]	0.9641	0.0163	0.1676 [0.1426; 0.1901]	0.9825	0.0698
**50 °C**	0.1743 [0.1675; 0.1811]	0.9988	0.0187	0.1776 [0.1700; 0.1853]	0.9986	0.0206	0.0065 [0.0053; 0.0078]	0.9461	0.0119	0.0088 [0.0078; 0.0099]	0.9765	0.0102	0.1197 [0.1125; 0.1271]	0.9967	0.0270
**40 °C**	0.1228 [0.1191; 0.1264]	0.9992	0.0133	0.1315 [0.1260; 0.1368]	0.9985	0.0187	0.0052 [0.0040; 0.0064]	0.9099	0.0126	0.0060 [0.0053; 0.0067]	0.9769	0.0071	0.0742 [0.0636; 0.0854]	0.9836	0.0455
**25 °C**	0.0915 [0.0863; 0.0968]	0.9975	0.0194	0.1072 [0.1033; 0.1113]	0.9986	0.0163	N/A	N/A	N/A	N/A	N/A	N/A	0.0356 [0.0313; 0.0399]	0.9845	0.0273

R^2 is the coefficient of determination.
Sx,y is the standard error of the regression.
Both R^2 and Sx,y are two key goodness-of-fit measures for regression analysis.

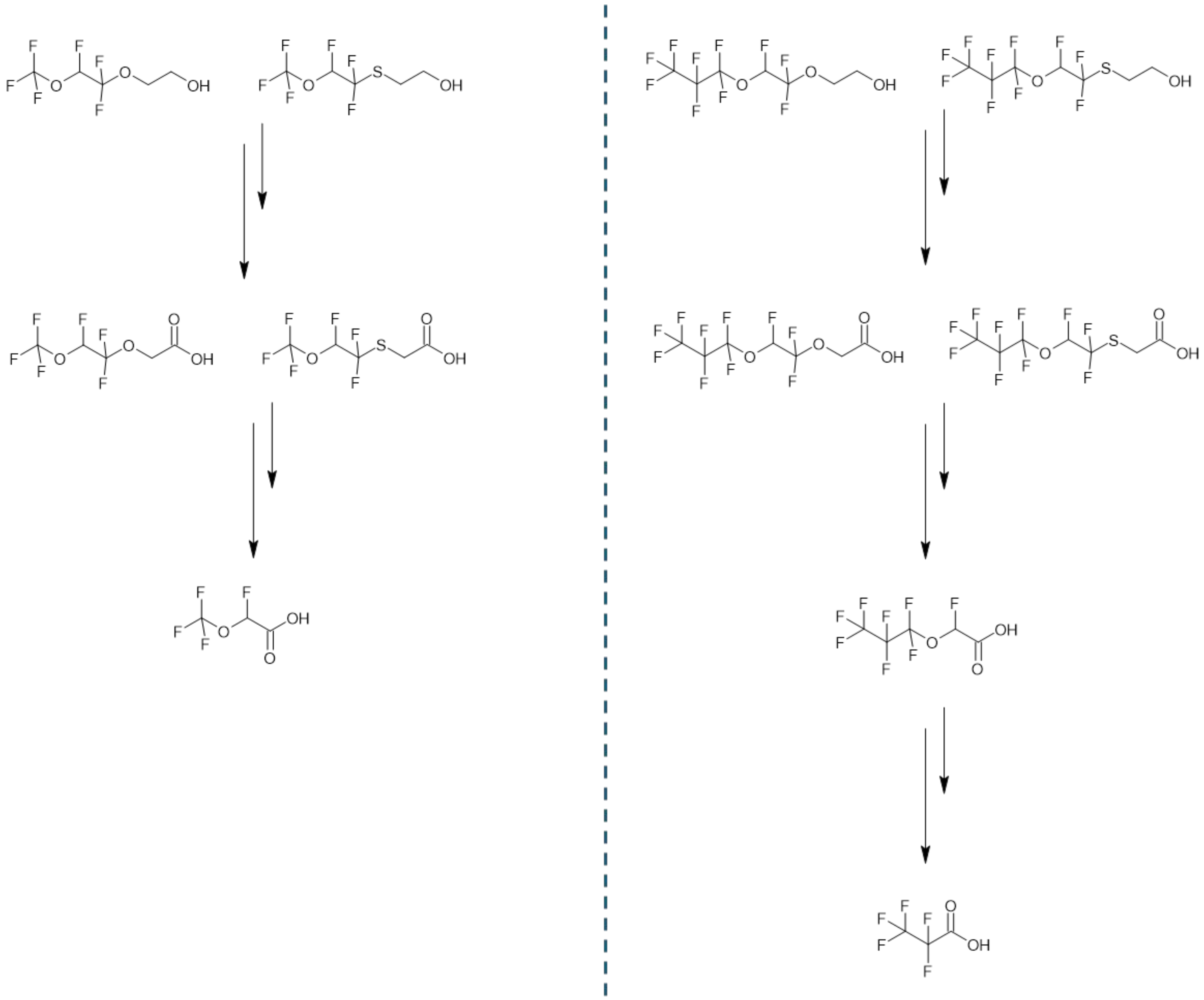

Figure S1: Proposed transformation pathway of the test chemicals in the 70 and 80 °C samples. Based on the studies of Licul-Kucera et al. [3] and Joudan & Mabury [5].

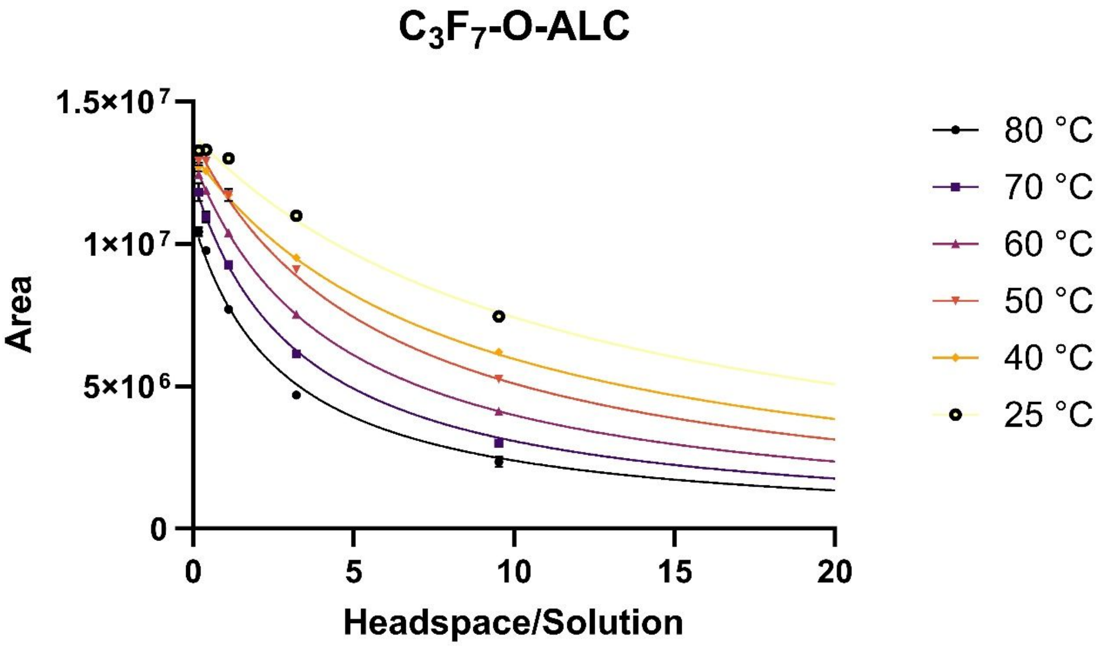

Figure S2: Plot used to the determination of K_{aw} for C_3F_7-O-ALC by direct approach.

Experimental data are shown by presenting the mean of *Area* values (data points) and their standard deviations (error bars) in terms of Headspace/Solution ratio at different temperatures (25-80 °C), while the solid lines indicate the result of the nonlinear regressions according to the Eq. 6.

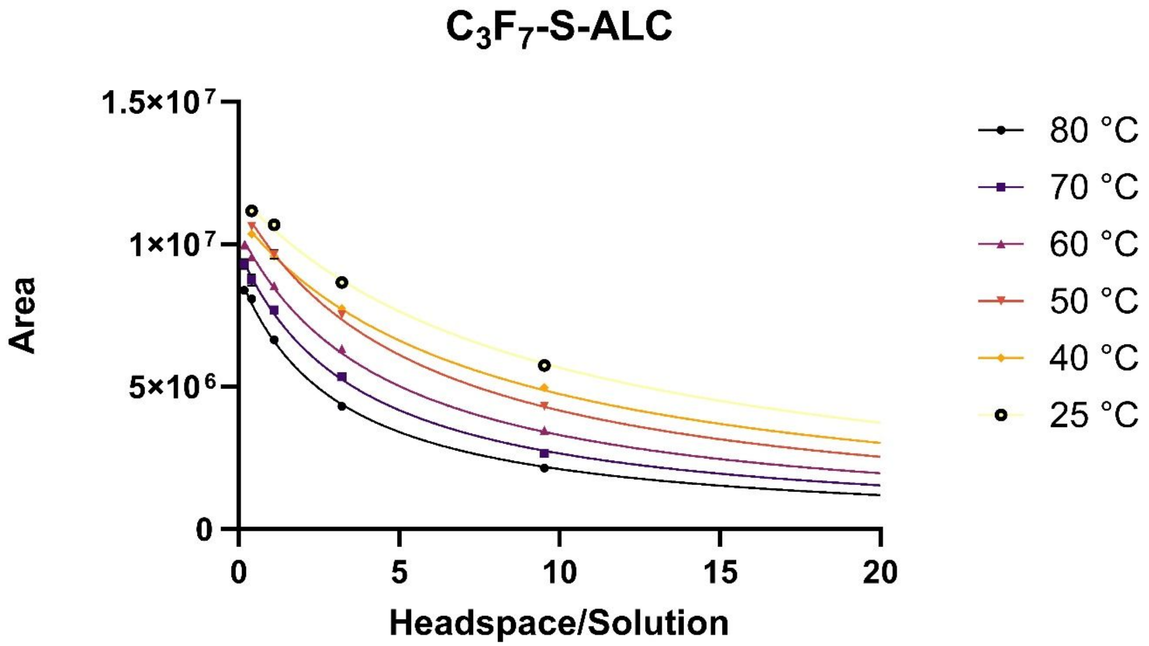

Figure S3: Plot used to the determination of K_{aw} for C_3F_7-S-ALC by direct approach.

Experimental data are shown by presenting the mean of *Area* values (data points) and their standard deviations (error bars) in terms of Headspace/Solution ratio at different temperatures (25-80 °C), while the solid lines indicate the result of the nonlinear regressions according to the Eq. 6.

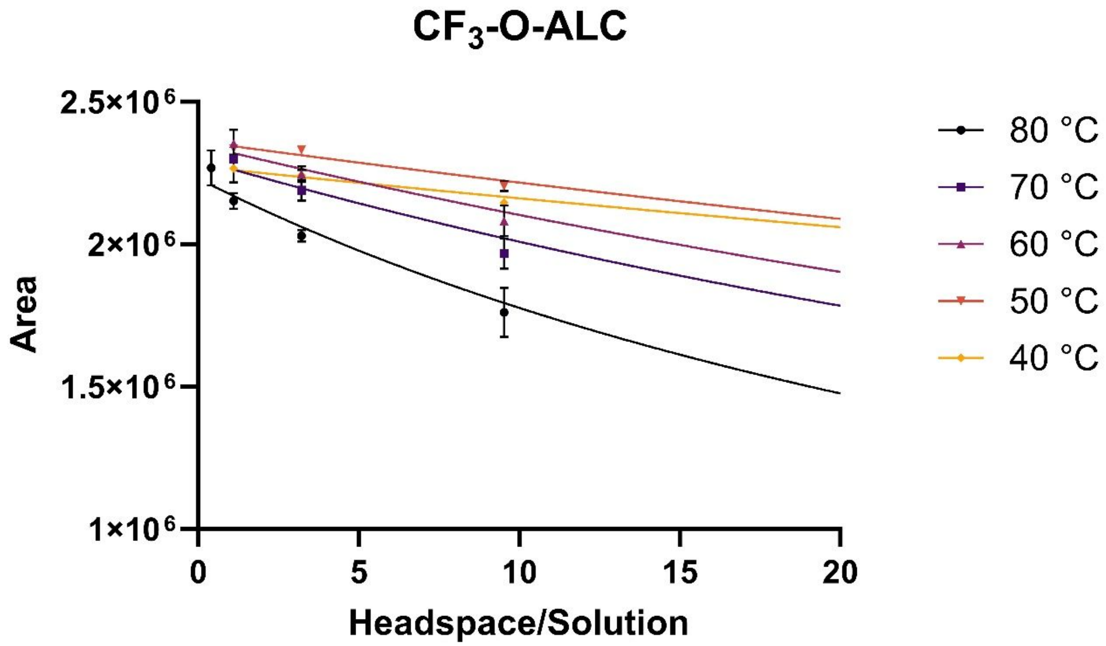

Figure S4: Plot used to the determination of K_{aw} for CF_3-O-ALC by direct approach.

Experimental data are shown by presenting the mean of *Area* values (data points) and their standard deviations (error bars) in terms of Headspace/Solution ratio at different temperatures (25-80 °C), while the solid lines indicate the result of the nonlinear regressions according to the Eq. 6.

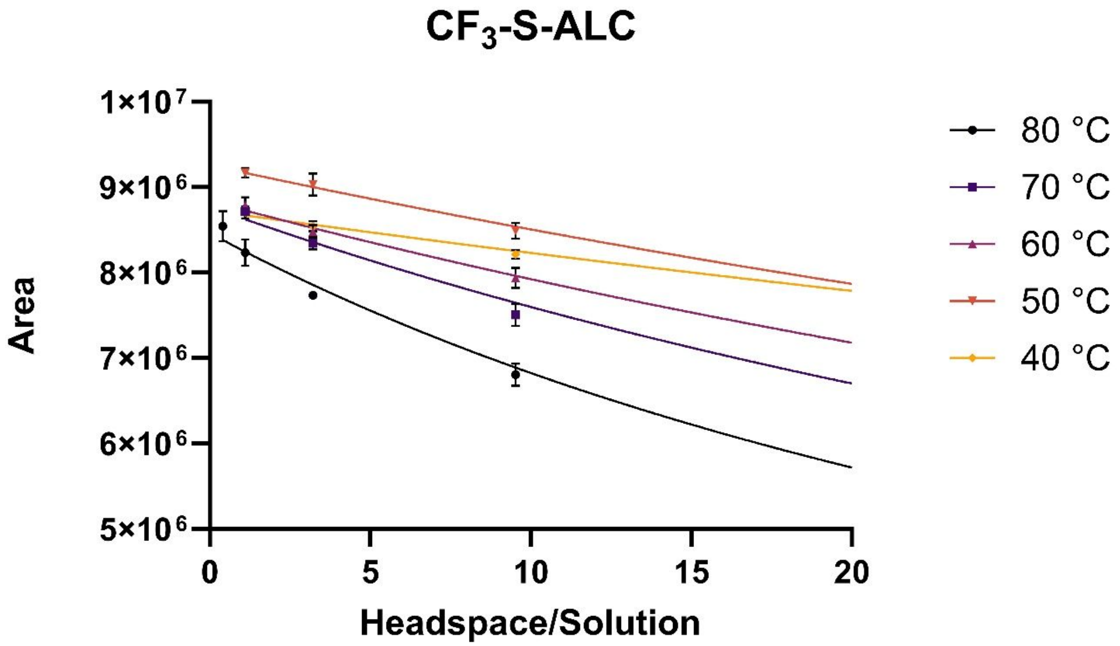

Figure S5: Plot used to the determination of K_{aw} for CF_3-S-ALC by direct approach.

A) Experimental data are shown by presenting the mean of *Area* values (data points) and their standard deviations (error bars) in terms of Headspace/Solution ratio at different temperatures (25-80 °C), while the solid lines indicate the result of the nonlinear regressions according to the Eq. 6.

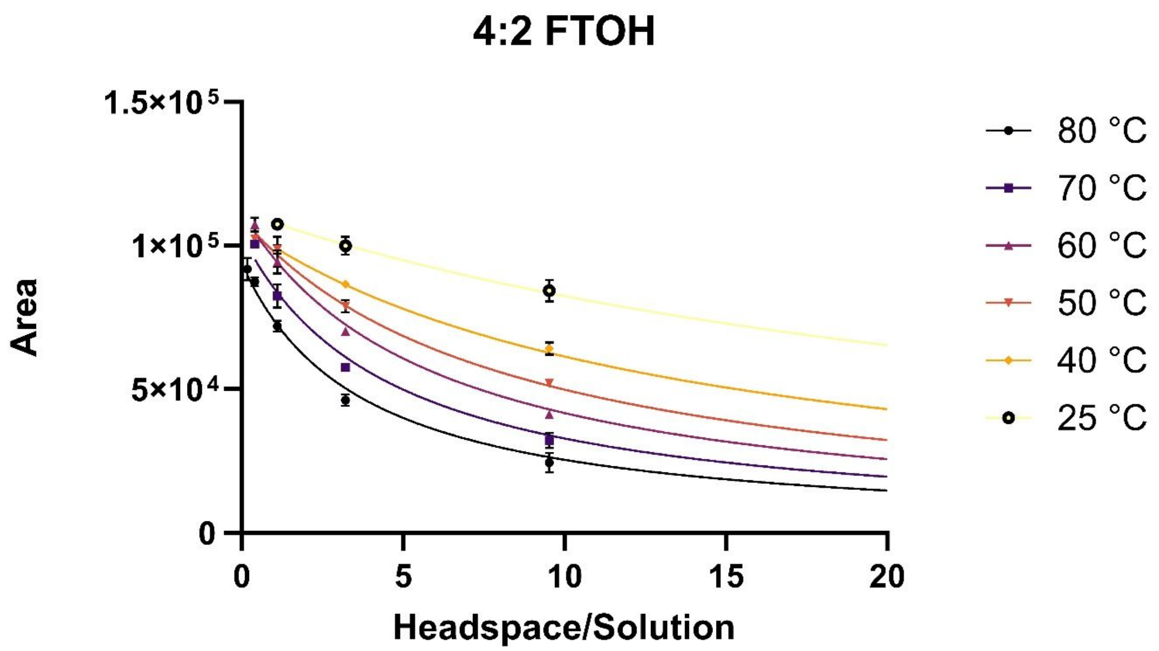

Figure S6: Plot used to the determination of K_{aw} for 4:2 FTOH by direct approach.

Experimental data are shown by presenting the mean of *Area* values (data points) and their standard deviations (error bars) in terms of Headspace/Solution ratio at different temperatures (25-80 °C), while the solid lines indicate the result of the nonlinear regressions according to the Eq. 6.

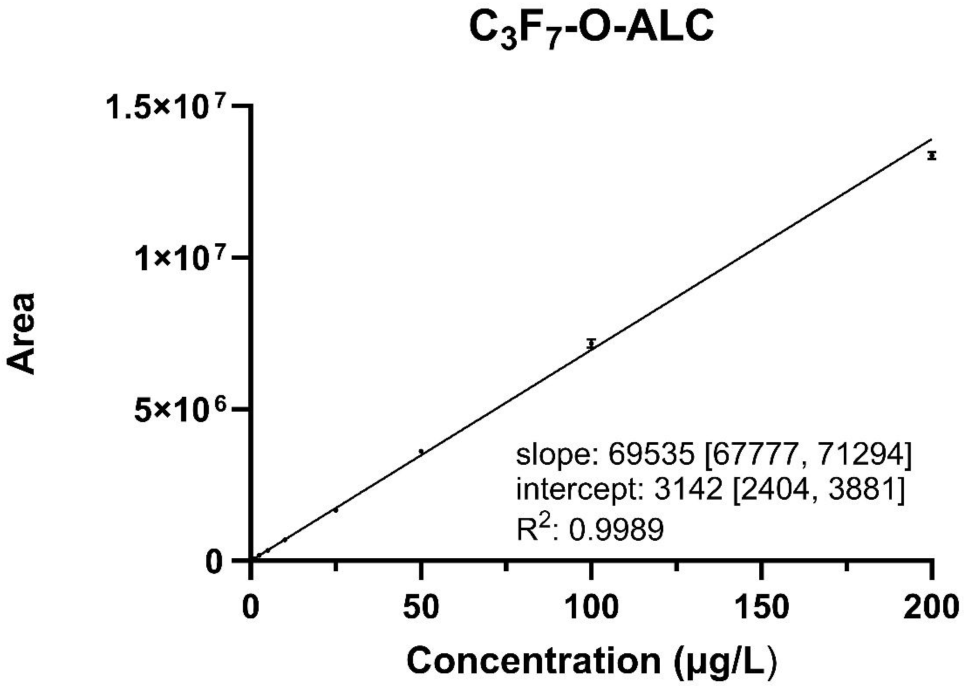

Figure S7: Linearity range of C_3F_7-O-ALC. Experimental data are shown by presenting the *Area*s (data points) measured by LC-MS and their standard deviations (error bars) in terms of *concentration* (µg/L), while the solid lines indicate the result of the linear regression. The mean values of slope and intercept of the linear with 95% confidence intervals (CI) (mean [95% CI], as well as the coefficient of determination (R^2) are also presented.

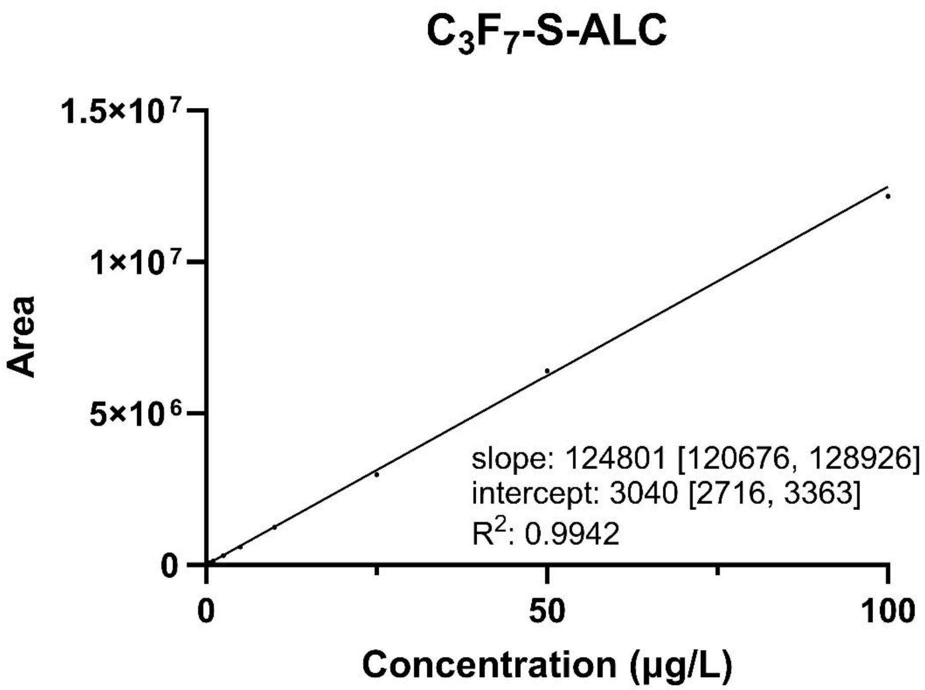

Figure S8: Linearity range of C_3F_7-S-ALC. Experimental data are shown by presenting the *Area*s (data points) measured by LC-MS and their standard deviations (error bars) in terms of *concentration* (µg/L), while the solid lines indicate the result of the linear regression. The mean values of slope and intercept of the linear with 95% confidence intervals (CI) (mean [95% CI], as well as the coefficient of determination (R^2) are also presented.

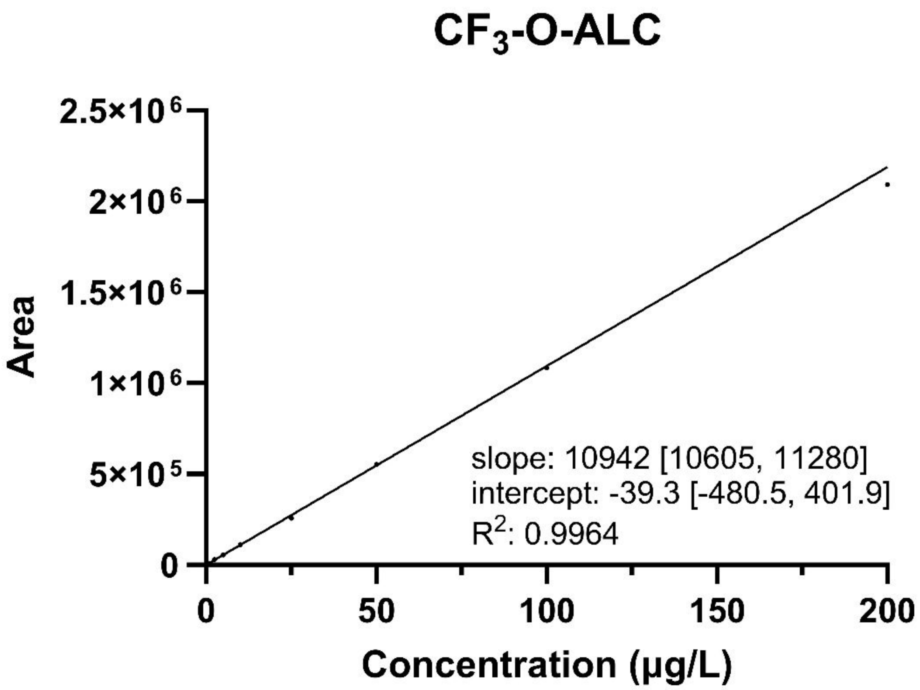

Figure S9: Linearity range of CF_3-O-ALC. Experimental data are shown by presenting the *Area*s (data points) measured by LC-MS and their standard deviations (error bars) in terms of *concentration* (µg/L), while the solid lines indicate the result of the linear regression. The mean values of slope and intercept of the linear with 95% confidence intervals (CI) (mean [95% CI], as well as the coefficient of determination (R^2) are also presented.

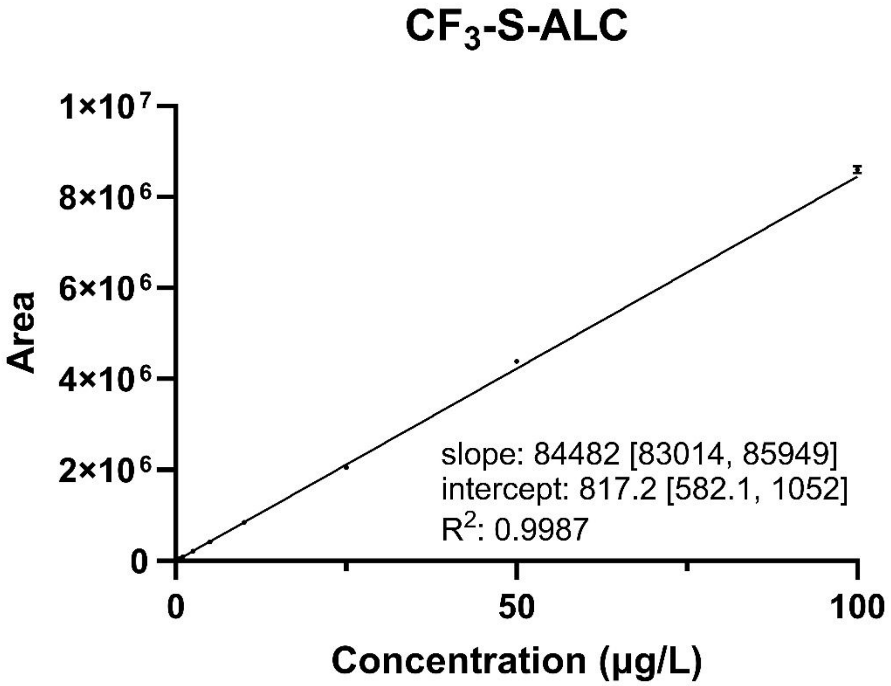

Figure S10: Linearity range of CF_3-S-ALC. Experimental data are shown by presenting the *Area*s (data points) measured by LC-MS and their standard deviations (error bars) in terms of *concentration* (μg/L), while the solid lines indicate the result of the linear regression. The mean values of slope and intercept of the linear with 95% confidence intervals (CI) (mean [95% CI], as well as the coefficient of determination (R^2) are also presented.

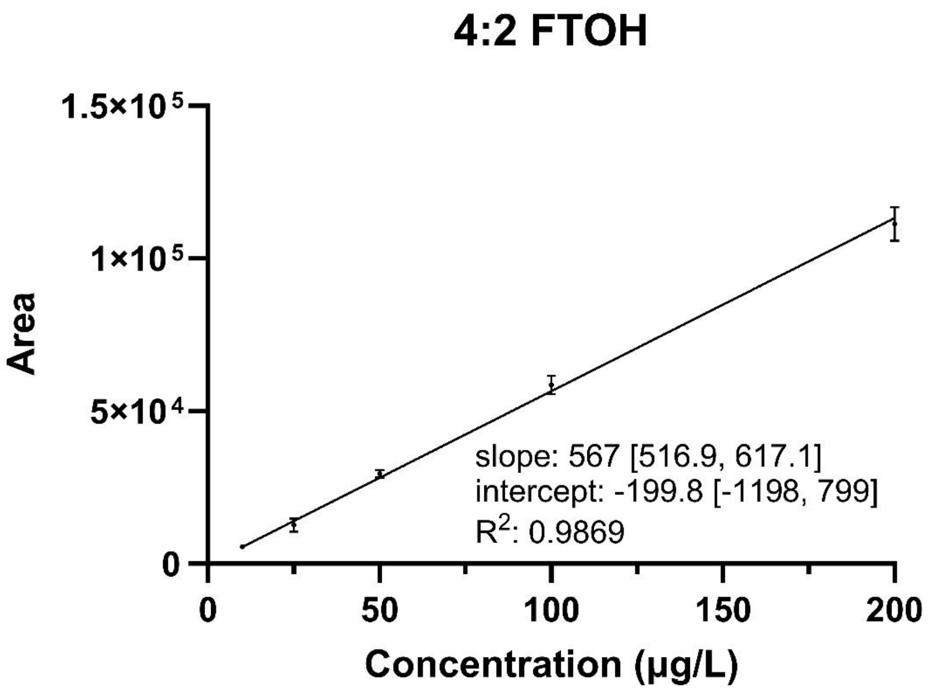

Figure S11: Linearity range of 4:2 FTOH. Experimental data are shown by presenting the *Area*s (data points) measured by LC-MS and their standard deviations (error bars) in terms of *concentration* (µg/L), while the solid lines indicate the result of the linear regression. The mean values of slope and intercept of the linear with 95% confidence intervals (CI) (mean [95% CI], as well as the coefficient of determination (R^2) are also presented.

References

(1) Berger, U.; Langlois, I.; Oehme, M.; Kallenborn, R. Comparison of Three Types of Mass Spectrometer for High-Performance Liquid Chromatography/Mass Spectrometry Analysis of Perfluoroalkylated Substances and Fluorotelomer Alcohols. *Eur. J. Mass Spectrom.* **2004**, *10*, 579–588. https://doi.org/10.1255/ejms.679.

(2) Gremmel, C.; Frömel, T.; Knepper, T. P. HPLC–MS/MS Methods for the Determination of 52 Perfluoroalkyl and Polyfluoroalkyl Substances in Aqueous Samples. *Anal. Bioanal. Chem.* **2016**, *409* (6), 1643–1655. https://doi.org/10.1007/s00216-016-0110-z.

(3) Licul-Kucera, V.; Frömel, T.; Krusa, M.; van Wezel, A. P.; Knepper, T. P. Finding a Way out? Comprehensive Biotransformation Study of Novel Fluorinated Surfactants. *Chemosphere* **2023**, *339* (139563). https://doi.org/10.1016/j.chemosphere.2023.139563.

(4) Wu, Y.; Chang, V. W.-C. The Effect of Surface Adsorption and Molecular Geometry on the Determination of Henry's Law Constants for Fluorotelomer Alcohols. *J. Chem. Eng. Data* **2011**, *56*, 3442–3448.

(5) Joudan, S.; Mabury, S. A. Aerobic Biotransformation of a Novel Highly Functionalized Polyfluoroether-Based Surfactant Using Activated Sludge from a Wastewater Treatment Plant. *Environ. Sci. Process. Impacts* **2022**, *24* (1), 62–71. https://doi.org/10.1039/d1em00358e.

(6) EAS-E Suite (Ver.0.97 - BETA, Release June, 2023). Www.Eas-e-Suite.Com. Accessed [20-09-2024]. Developed by ARC Arnot Research and Consulting Inc., Toronto, ON, Canada. **2024**, 2024.

(7) Brown, T. N.; Arnot, J. A.; Wania, F. Iterative Fragment Selection: A Group Contribution Approach to Predicting Fish Biotransformation Half-Lives.

Environ. Sci. Technol. **2012**, *46*, 8253–8260.

(8) Endo, S.; Goss, K.-U. Applications of Polyparameter Linear Free Energy Relationships in Environmental Chemistry. *Environ. Sci. Technol.* **2014**, *48*, 12477–12491.

(9) Ulrich, N.; Endo, S.; Brown, T. N.; Watanabe, N.; Bronner, G.; Abraham, M. H.; Goss, K.-U. *UFZ-LSER database v 3.2.1 [Internet], Leipzig, Germany, Helmholtz Centre for Environmental Research-UFZ. 2017 [accessed on 02.04.2024]. Available from http://www.ufz.de/lserd.*

(10) Brown, T. N. QSPRs for Predicting Equilibrium Partitioning in Solvent–Air Systems from the Chemical Structures of Solutes and Solvents. *J. Solution Chem.* **2022**, *51*, 1101–1132. https://doi.org/10.1007/s10953-022-01162-2.

(11) Mansouri, K.; Grulke, C. M.; Judson, R. S.; Williams, A. J. OPERA Models for Predicting Physicochemical Properties and Environmental Fate Endpoints. *J. Cheminform.* **2018**, *10* (10). https://doi.org/10.1186/s13321-018-0263-1.

(12) Hine, J.; Mookerjee, P. K. The Intrinsic Hydrophilic Character of Organic Compounds. Correlations in Terms of Structural Contributions. *J. Org. Chem.* **1975**, *40*, 292–298.

(13) Endo, S.; Hammer, J.; Matsuzawa, S. Experimental Determination of Air/Water Partition Coefficients for 21 Per- and Polyfluoroalkyl Substances Reveals Variable Performance of Property Prediction Models. *Environ. Sci. Technol.* **2023**, *57*, 8406–8413. https://doi.org/10.1021/acs.est.3c02545.

(14) Eckert, F.; Klamt, A. Fast Solvent Screening via Quantum Chemistry: The COSMO-RS Approach. *AIChE J.* **2002**, *48*, 369–385. https://doi.org/10.1002/aic.690480220/full.

(15) Klamt, A. Conductor-like Screening Model for Real Solvents: A New

Approach to the Quantitative Calculation of Solvation Phenomena. *J. Phys. Chem. A* **1995**, *99*, 2224–2235.

(16) Folkerson, A. P.; Schneider, S. R.; Abbatt, J. P. D.; Mabury, S. A. Avoiding Regrettable Replacements: Can the Introduction of Novel Functional Groups Move PFAS from Recalcitrant to Reactive? *Environ. Sci. Technol.* **2023**, *57* (44), 17032–17041. https://doi.org/10.1021/acs.est.3c06232.

(17) Ellis, D. A.; Martin, J. W.; Mabury, S. A. Atmospheric Lifetime of Fluorotelomer Alcohols. *Environ. Sci. Technol.* **2003**, *37*, 3816–3820.

(18) Joudan, S.; Orlando, J. J.; Tyndall, G. S.; Furlani, T. C.; Young, C. J.; Mabury, S. A. Atmospheric Fate of a New Polyfluoroalkyl Building Block, C3F7OCHFCF2SCH2CH2OH. *Environ. Sci. Technol.* **2022**, *56* (10), 6027–6035. https://doi.org/10.1021/acs.est.0c07584.
